# Supplementary material for: Pharmacokinetic-Pharmacodynamic Modelling of Systemic IL13 Blockade by Monoclonal Antibody Therapy: A Free Assay Disguised as Total
Source: Pharmaceutics. 2021 Apr 9;13(4):519. doi: 10.3390/pharmaceutics13040519 (PMC8069034; doi:10.3390/pharmaceutics13040519)
Supplement: Supplementary file 1 [file pharmaceutics-13-00519-s001.pdf]

pharmaceutics

MDPI

Supplementary Materials: Pharmacokinetic-Pharmacodynamic Modelling of Systemic IL13 Blockade by Monoclonal Antibody Therapy: A Free Assay Disguised as Total

Jana Kubackova, Ondrej Holas, Jarmila Zbytovska, Barbora Vranikova, Guanghong Zeng, Petr Pavek and Anette Mullertz

Supplementary information

1. Goodness of Fit Plots

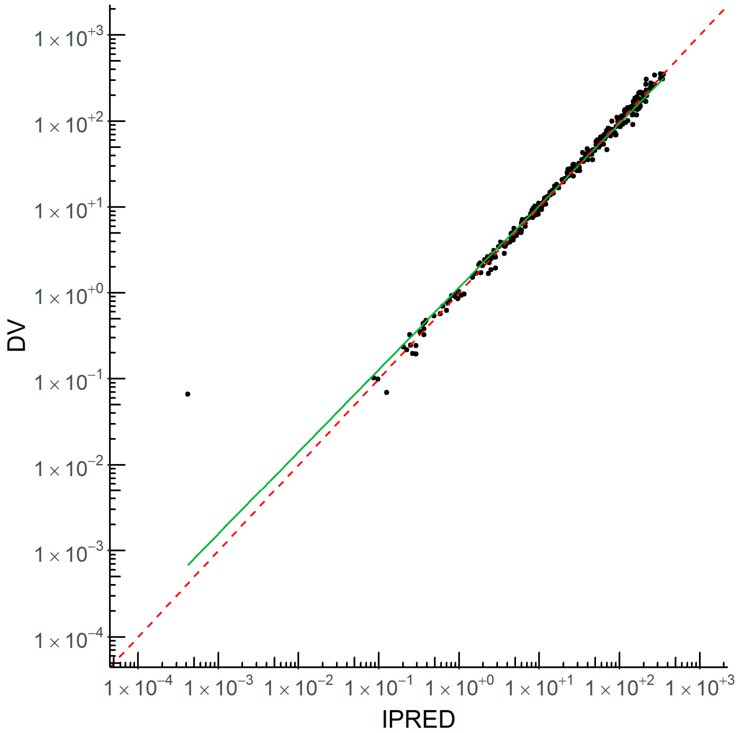

Figure S1. IPRED vs DV for concentrations of MEDI7836 in healthy human volunteers.

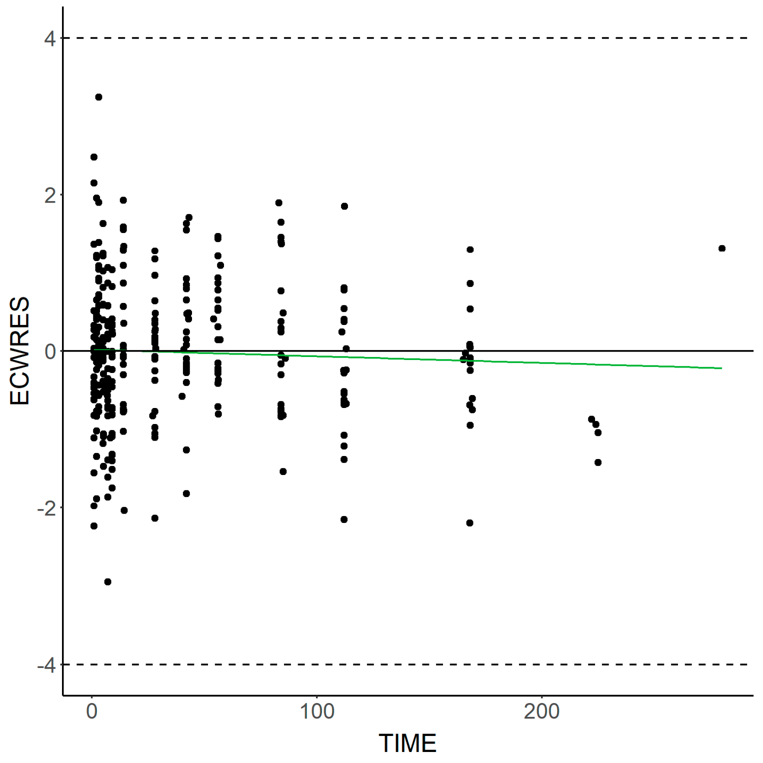

Figure S2. CWRES vs Time for concentrations of MEDI7836 in healthy human volunteers.

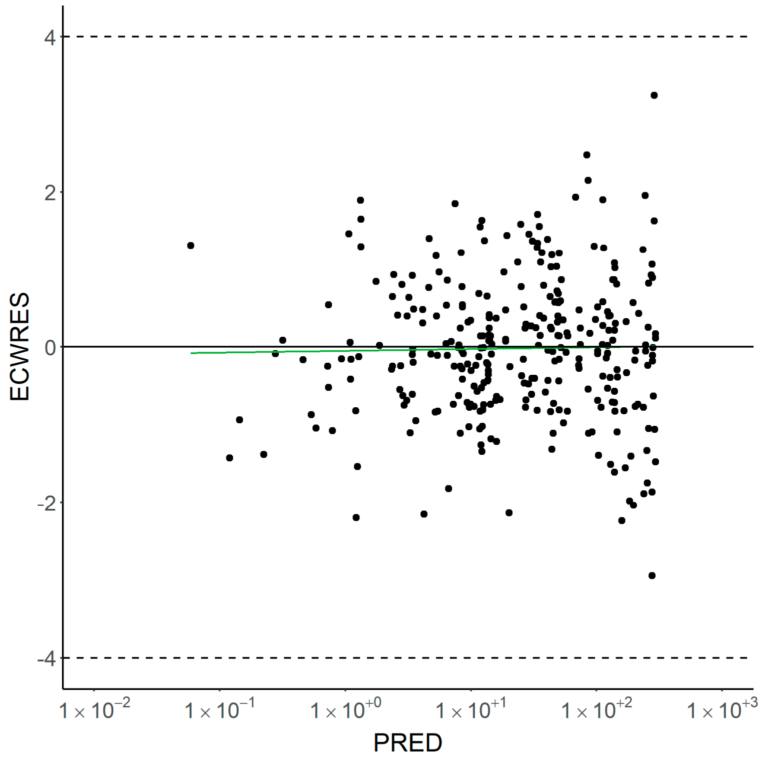

Figure S3. CWRES vs PRED for concentrations of MEDI7836 in healthy human volunteers.

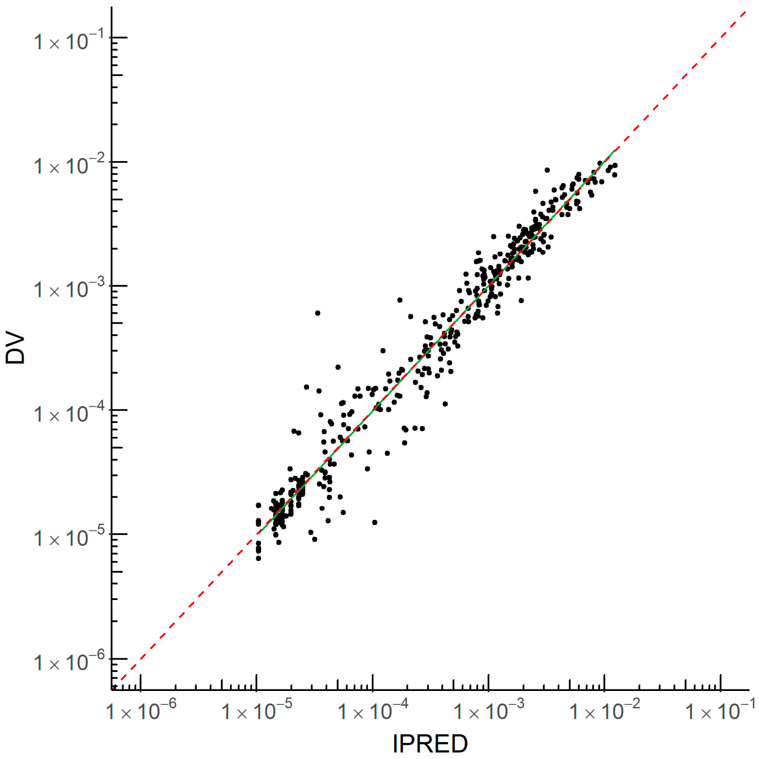

Figure S4. DV vs IPRED for concentrations of MEDI7836-IL13 complex in healthy human volunteers.

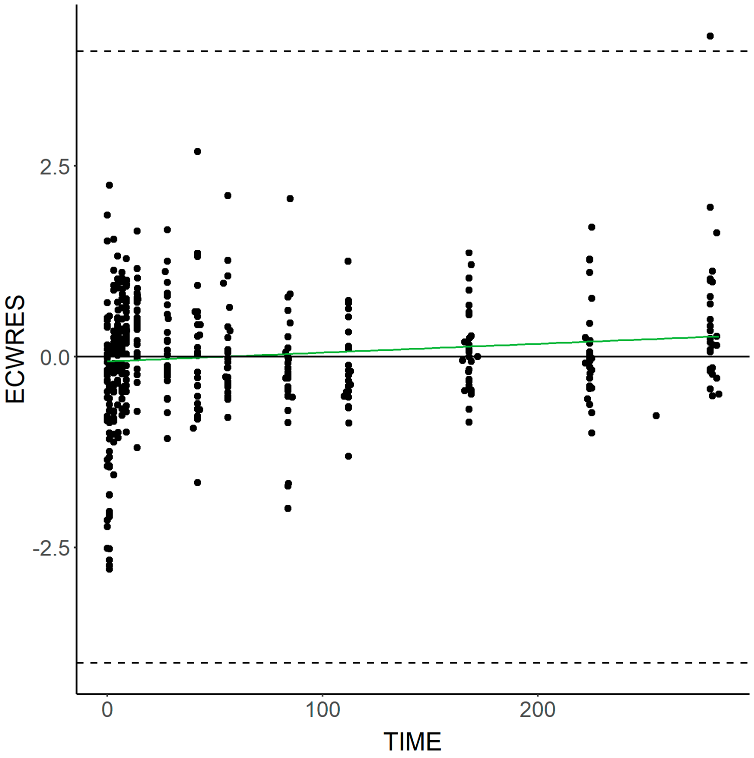

Figure S5. CWRES vs. Time for concentrations of IL13 and MEDI7836-IL13 complex in healthy human volunteers.

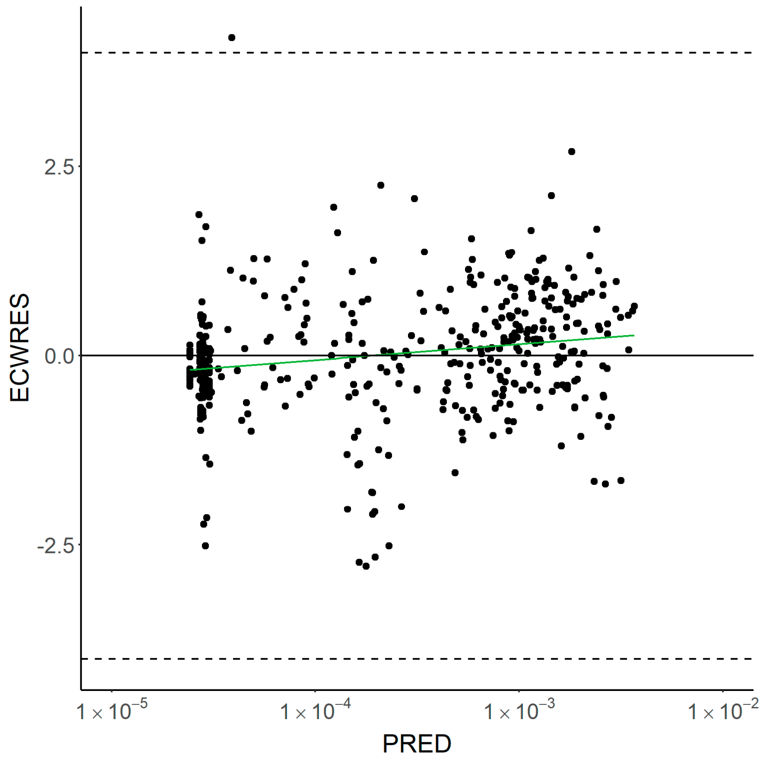

Figure S6. CWRES vs PRED for concentrations of IL13 and MEDI7836-IL13 complex in healthy human volunteers.

2. NONMEM PK Script

```
$PROBLEM       MEDI7836 popPK
$INPUT         SUBJID ID TIME VISITNUM MAMT AMT CMT MDV EDV LNDV NMDV DV
               DVFLAG DOSE AGE WT SEXN RACEN ZADY ZAORRES ADAB ADAT ADA2
               ADA3 SWAP FLAG1 FLAG2 IGN
$DATA          NM_MEDI7836_IL13_LNDV_outfrom49_21Feb19.csv IGNORE=@
               IGNORE(ID.EQ.1)
$SUBROUTINE ADVAN13 TOL=5
$MODEL         COMP=(DEPOT) ;#1 Absorption
               COMP=(CENTRAL) ;#2 Central Compartment (obs)
               COMP=(PERI) ;#3 Peripheral Compartment
               COMP=(IL13) ;#4 Free IL13
               COMP=(CMP1) ;#5 IL13:MEDI7836 Complex Central
               COMP=(CMP2) ;#5 IL13:MEDI7836 Complex Peripheral
$PK
;Covariates
SHIFT = 0
IF(ADA3.EQ.1) SHIFT =THETA(15)
;PK
TVCL =THETA(3)*(1+SHIFT)
      MU_3=LOG(TVCL)
      CL=TVCL*EXP(ETA(3))
```

```
TVV2 =THETA(4)
    MU_4=LOG(TVV2)
    V2=TVV2*EXP(ETA(4))
TVKA =THETA(5)
    MU_5=LOG(TVKA)
    KA=TVKA*EXP(ETA(5))
TVV3 =THETA(1)
    MU_1=LOG(TVV3)
    V3=TVV3*EXP(ETA(1))
TVQ =THETA(2)
    MU_2=LOG(TVQ)
    Q=TVQ*EXP(ETA(2))
K=CL/V2
S2=V2
K23=Q/V2
K32=Q/V3
AUC=AMT/CL
; -------- PD -------- ;
TVKIN = THETA(6)
      MU_6 = LOG(TVKIN)
      KIN = TVKIN*EXP(ETA(6))
TVKOUT = THETA(7)
      MU_7 = LOG(TVKOUT)
      KOUT = TVKOUT*EXP(ETA(7))
TVFR = THETA(8)
      MU_8 = LOG(TVFR)
      PHI = LOG(TVFR/(1-TVFR))
      FR = EXP(PHI+ETA(8)) / (1+EXP(PHI+ETA(8)))
TVCXV2 = THETA(9)
      MU_9 = LOG(TVCXV2)
      CXV2 = TVCXV2*EXP(ETA(9))
KON    = THETA(10)
KOFF = THETA(11)
BASE = KIN/KOUT
A_0(4) = BASE
A_0(5) = 0
A_0(6) = 0
$DES
; -------- PK -------- ;
C2 = A(2)/V2
C3 = A(3)/V3
ABSO = KA*A(1)
```

```
DIST = Q*(C2 - C3)
ELIM = CL*C2
DADT(1) = - ABSO
DADT(2) =      ABSO - DIST - ELIM
DADT(3) =                   DIST
; -------- PD -------- ;
IL_PROD = KIN
IL_ELIM = KOUT*A(4)
CX2         = A(5)/CXV2
CX3         = A(6)/V3
CX_PROD = KON*A(4)*C2
CX_DISS = KOFF*A(5)
CX_ELIM = CL*CX2
CX_DIST = Q*(CX2-CX3)
DADT(4) = IL_PROD - IL_ELIM + CX_DISS - CX_PROD
DADT(5) =                                 - CX_DISS + CX_PROD - CX_ELIM -CX_DIST
DADT(6) = CX_DIST
$ERROR
CP = A(2)/V2                    ; concentration in nmol/L
CX = A(5)/V2                                    ; concentration in nmol/L
IL13 = A(4)/V2                             ; concentration in nmol/L
STRT = (DOSE+CMT)
ADD    = THETA(12)
PROP = THETA(13)/100
ILPROP = THETA(14)/100
PPK = 0
PPD = 0
IF(CMT.EQ.2) PPK = 1
IF(CMT.EQ.4) PPD = 1

IPRED = 0
IF(CMT.EQ.2) IPRED = F
IF(CMT.EQ.4) IPRED = LOG(IL13 + FR*CX)
IRES    = DV - IPRED
WPK      = SQRT(ADD**2 + PROP**2*IPRED**2)*PPK
WPD      = ILPROP*PPD
IWRES = IRES / (WPK+WPD)
IF(CMT.EQ.2) Y = IPRED + WPK*EPS(1)
IF(CMT.EQ.4) Y = IPRED * (1 + WPD*EPS(2))

$THETA    8.18784 FIX ; #1 V3/F (L)
$THETA    0.810915 FIX ; #2 Q (1/d)
```

```
$THETA    0.437872 FIX ; #3 CL/F (L/d)
$THETA    2.89047 FIX ; #4 V2/F (L)
$THETA    0.157075 FIX ; #5 KA (1/d)
$THETA    (0,0.0111923) ; #6 KIN (nmol/d)
$THETA    (0,149.364) ; #7 KOUT (1/d)
$THETA    (0,0.0778307) ; #8 Cx fraction
$THETA    (0,7.2956) ; #8 CXV2 Complex central vol (L)
$THETA    138.24 FIX ; #10 KON (1/d*nM)
$THETA    0.69 FIX ; #11 KOFF (1/*nM)
$THETA    0.0535482 FIX ; #12 PK RSV (ug/L)
$THETA    12.8717 FIX ; #13 PK RSV (%)
$THETA    (0,5.78824) ; #14 PD-RSV (%)
$THETA    0.722631 FIX ; #15 SHIFT
$OMEGA    0.207367 FIX ;    #1 BSV_V3
$OMEGA    0.618521 FIX ;     #2 BSV_Q
$OMEGA    0.264773    FIX    ;    #3 BSV_CL
$OMEGA    0.42625    FIX    ;    #4 BSV_V2
$OMEGA    0    FIX    ;    #5 BSV_KA
$OMEGA    0.0225    FIX    ; #6 BSV_KIN
$OMEGA    0.292198    ; #7 BSV_KOUT
$OMEGA    1.27288    ;    #8 BSV_FR
$OMEGA    0.0225    FIX    ; #9 BSV_CXVol2
$SIGMA    1    FIX
$SIGMA    1    FIX
$ESTIMATION METHOD=SAEM INTER NBURN=3000 ISAMPLE=2 PRINT=5 NITER=1000
            CTYPE=3 CITER=10 GRD=DDDDDDDDSS
$ESTIMATION METHOD=IMP INTER EONLY=1 ISAMPLE=3000 NITER=10 MAPITER=0
            CITER=10 CALPHA=0.05 GRD=DDDDDDDDSS
$COVARIANCE PRINT=E UNCONDITIONAL
$TABLE        SUBJID ID TIME EPRED IPRED ERES EWRES ECWRES VISITNUM DOSE
            EVID AMT CMT IL13 CP CX STRT NOPRINT ONEHEADER
            FORMAT=s1PE17.10 FILE=sdtab51
$TABLE        SUBJID ID CL V2 Q V3 KA AUC KIN KOUT BASE ETA1 ETA2 ETA3
            ETA4 ETA5 ETA6 ETA7 ETA8 ETA9 FIRSTONLY NOAPPEND NOPRINT
            FORMAT=s1PE17.10 FILE=patab51
$TABLE        SUBJID ID AGE WT FIRSTONLY NOAPPEND NOPRINT ONEHEADER
            FORMAT=s1PE17.10 FILE=cotab51
$TABLE        SUBJID ID SEXN RACEN ZADY ZAORRES ADAB ADAT ADA2 ADA3 DOSE
            FIRSTONLY NOAPPEND NOPRINT ONEHEADER FORMAT=s1PE17.10
            FILE=catab51
```

3. NONMEM PD Script

```
$PROBLEM      MEDI7836 popPK
$INPUT        SUBJID ID TIME VISITNUM MAMT AMT CMT MDV EDV LNDV NMDV DV
              DVFLAG DOSE AGE WT SEXN RACEN ZADY ZAORRES ADAB ADAT ADA2
              ADA3 SWAP FLAG1 FLAG2 IGN
$DATA         NM_MEDI7836_IL13_LNDV_outfrom49_21Feb19.csv IGNORE=@
              IGNORE(ID.EQ.1)
$SUBROUTINE ADVAN13 TOL=5
$MODEL        COMP=(DEPOT) ;#1 Absorption
              COMP=(CENTRAL) ;#2 Central Compartment (obs)
              COMP=(PERI) ;#3 Peripheral Compartment
              COMP=(IL13) ;#4 Free IL13
              COMP=(CMP1) ;#5 IL13:MEDI7836 Complex Central
              COMP=(CMP2) ;#5 IL13:MEDI7836 Complex Peripheral
$PK
;Covariates
SHIFT = 0
IF(ADA3.EQ.1) SHIFT =THETA(15)
;PK
TVCL =THETA(3)*(1+SHIFT)
    MU_3=LOG(TVCL)
    CL=TVCL*EXP(ETA(3))
TVV2 =THETA(4)
    MU_4=LOG(TVV2)
    V2=TVV2*EXP(ETA(4))
TVKA =THETA(5)
    MU_5=LOG(TVKA)
    KA=TVKA*EXP(ETA(5))
TVV3 =THETA(1)
    MU_1=LOG(TVV3)
    V3=TVV3*EXP(ETA(1))
TVQ =THETA(2)
    MU_2=LOG(TVQ)
    Q=TVQ*EXP(ETA(2))
K=CL/V2
S2=V2
K23=Q/V2
K32=Q/V3
AUC=AMT/CL
; -------- PD -------- ;
TVKIN = THETA(6)
      MU_6 = LOG(TVKIN)
```

```
        KIN = TVKIN*EXP(ETA(6))
TVKOUT = THETA(7)
        MU_7 = LOG(TVKOUT)
        KOUT = TVKOUT*EXP(ETA(7))
TVFR = THETA(8)
        MU_8 = LOG(TVFR)
        PHI = LOG(TVFR/(1-TVFR))
        FR = EXP(PHI+ETA(8)) / (1+EXP(PHI+ETA(8)))
TVCXV2 = THETA(9)
        MU_9 = LOG(TVCXV2)
        CXV2 = TVCXV2*EXP(ETA(9))
KON    = THETA(10)
KOFF = THETA(11)
BASE = KIN/KOUT
A_0(4) = BASE
A_0(5) = 0
A_0(6) = 0
$DES
; -------- PK -------- ;
C2 = A(2)/V2
C3 = A(3)/V3
ABSO = KA*A(1)
DIST = Q*(C2 - C3)
ELIM = CL*C2
DADT(1) = - ABSO
DADT(2) =     ABSO - DIST - ELIM
DADT(3) =                 DIST
; -------- PD -------- ;
IL_PROD = KIN
IL_ELIM = KOUT*A(4)
CX2         = A(5)/CXV2
CX3         = A(6)/V3
CX_PROD = KON*A(4)*C2
CX_DISS = KOFF*A(5)
CX_ELIM = CL*CX2
CX_DIST = Q*(CX2-CX3)
DADT(4) = IL_PROD - IL_ELIM + CX_DISS - CX_PROD
DADT(5) =                          - CX_DISS + CX_PROD - CX_ELIM -CX_DIST
DADT(6) = CX_DIST
$ERROR
CP = A(2)/V2                 ; concentration in nmol/L
CX = A(5)/V2                              ; concentration in nmol/L
```

```
IL13 = A(4)/V2                    ; concentration in nmol/L
STRT = (DOSE+CMT)
ADD   = THETA(12)
PROP = THETA(13)/100
ILPROP = THETA(14)/100
PPK = 0
PPD = 0
IF(CMT.EQ.2) PPK = 1
IF(CMT.EQ.4) PPD = 1

IPRED = 0
IF(CMT.EQ.2) IPRED = F
IF(CMT.EQ.4) IPRED = LOG(IL13 + FR*CX)
IRES   = DV - IPRED
WPK     = SQRT(ADD**2 + PROP**2*IPRED**2)*PPK
WPD     = ILPROP*PPD
IWRES = IRES / (WPK+WPD)
IF(CMT.EQ.2) Y = IPRED + WPK*EPS(1)
IF(CMT.EQ.4) Y = IPRED * (1 + WPD*EPS(2))

$THETA   7.84708 FIX ; #1 V3/F (L)
$THETA   0.864868 FIX ; #2 Q (1/d)
$THETA   0.438604 FIX ; #3 CL/F (L/d)
$THETA   3.0941 FIX ; #4 V2/F (L)
$THETA   0.167657 FIX ; #5 KA (1/d)
$THETA   (0,0.011423) ; #6 KIN (nmol/d)
$THETA   (0,110.086) ; #7 KOUT (1/d)
$THETA   (0,0.107495) ; #8 Cx fraction
$THETA   (0,3.09) ; #8 CXV2 Complex central vol (L)
$THETA   138.24 FIX ; #10 KON (1/d*nM)
$THETA   0.69 FIX ; #11 KOFF (1/*nM)
$THETA   0.0537212 FIX ; #12 PK RSV (ug/L)
$THETA   12.6818 FIX ; #13 PK RSV (%)
$THETA   (0,6.06288) ; #14 PD-RSV (%)
$THETA   0.735988 FIX ; #15 SHIFT
$OMEGA   BLOCK(2) FIX
 0.254214   ;   #1 BSV_V3
 0.199976 0.387745   ;     #2 BSV_Q
$OMEGA   0.266036   FIX   ;   #3 BSV_CL
$OMEGA   0.40104   FIX   ;   #4 BSV_V2
$OMEGA   0.0225   FIX   ;   #5 BSV_KA
$OMEGA   0.0225   FIX   ; #6 BSV_KIN
```

```
$OMEGA    0.262356    ; #7 BSV_KOUT
$OMEGA    1.4567    ;    #8 BSV_FR
$OMEGA    0.0225 FIX    ;    #9 BSV_CXVol2
$SIGMA    1    FIX
$SIGMA    1    FIX
$ESTIMATION METHOD=SAEM INTER NBURN=3000 ISAMPLE=2 PRINT=5 NITER=1000
            CTYPE=3 CITER=10 GRD=DDDDDDDDSS
$ESTIMATION METHOD=IMP INTER EONLY=1 ISAMPLE=3000 NITER=10 MAPITER=0
            CITER=10 CALPHA=0.05 GRD=DDDDDDDDSS
$COVARIANCE PRINT=E UNCONDITIONAL
$TABLE       SUBJID ID TIME EPRED IPRED ERES EWRES ECWRES VISITNUM DOSE
            EVID AMT CMT IL13 CP CX STRT NOPRINT ONEHEADER FORMAT=s1PE17.10
            FILE=sdtab33
$TABLE       SUBJID ID CL V2 Q V3 KA AUC KIN KOUT BASE ETA1 ETA2 ETA3
            ETA4 ETA5 ETA6 ETA7 ETA8 FIRSTONLY NOAPPEND NOPRINT
            FORMAT=s1PE17.10 FILE=patab33
$TABLE       SUBJID ID AGE WT FIRSTONLY NOAPPEND NOPRINT ONEHEADER
            FORMAT=s1PE17.10 FILE=cotab33
$TABLE       SUBJID ID SEXN RACEN ZADY ZAORRES ADAB ADAT ADA2 ADA3 DOSE
            FIRSTONLY NOAPPEND NOPRINT ONEHEADER FORMAT=s1PE17.10
            FILE=catab33
```
